# Broadly Protective Antibody‐Like Vaccines Against Highly Pathogenic Coronaviruses

**DOI:** 10.1002/jmv.71008

**Published:** 2026-06-22

**Authors:** Assala Helal, Najwa D. Aljehani, Aishah Ghazwani, Reem M. Alsulaiman, Faris Alyami, Wesam H. Abdulaal, Asem Alsharef, Maimonah Alghanmi, Ayat Zawawi, Ala A. Azhari, Afnan Almehmadi, Jood Balula, Tarfa Altorki, Rowa Y. Alhabbab, Muhammad Yasir Khan, Khalid Alluhaybi, Aisha Alnami, Thikryat Neamatallah, Turki S. Abujamel, Mohamed A. Alfaleh, Anwar M. Hashem

**Affiliations:** ^1^ Vaccines and Immunotherapy Unit, King Fahd Medical Research Center King Abdulaziz University Jeddah Saudi Arabia; ^2^ Department of Pharmacology and Toxicology, Faculty of Pharmacy King Abdulaziz University Jeddah Saudi Arabia; ^3^ Department of Biochemistry, Faculty of Science King Abdulaziz University Jeddah Saudi Arabia; ^4^ Department of Medical Laboratory Technology, Faculty of Applied Medical Sciences King Abdulaziz University Jeddah Saudi Arabia; ^5^ Department of Clinical Microbiology and Immunology, Faculty of Medicine King Abdulaziz University Jeddah Saudi Arabia; ^6^ Department of Pharmaceutics, Faculty of Pharmacy King Abdulaziz University Jeddah Saudi Arabia; ^7^ Department of Pharmaceutical Chemistry, Faculty of Pharmacy King Abdulaziz University Jeddah Saudi Arabia

**Keywords:** antibodies, coronaviruses, Fc‐fusion, MERS‐CoV, SARS‐CoV‐2, vaccines

## Abstract

SARS‐CoV‐2 and MERS‐CoV are highly pathogenic and contagious coronaviruses. Despite intensive vaccination, SARS‐CoV‐2 continues to spread, and no FDA‐approved vaccines exist for MERS‐CoV; thus, more effective and safer vaccine options are needed. The receptor‐binding domain (RBD) of coronaviruses is a primary target of neutralizing antibodies (nAbs); therefore, we generated three bivalent IgG1 Fc‐fusion vaccines (BiVaxs) combining SARS‐CoV‐2 and MERS‐CoV RBDs. The BiVax_Fc‐Native_ vaccine comprises ^omicron's‐RBD^SARS‐CoV‐2 fused to the native Fc C‐terminus and ^RBD^MERS‐CoV to the N‐terminus. The BiVax_Fc‐Reverse_ vaccine has a native Fc, yet ^RBD^MERS‐CoV was fused to the C‐terminus, and the ^omicron's‐RBD^SARS‐CoV‐2 to the N‐terminus. The third was BiVax_Fc‐FcRn_, which is similar to BiVax_Fc‐Native_, although it contains MST‐HN Fc mutations (M252Y/S254T/T256E‐H433K/N434F) to enhance neonatal Fc receptor (FcRn) binding on antigen‐presenting cells (APCs). In mice, BiVax_Fc‐FcRn_ showed significantly higher immunogenicity than the other two forms. It induced robust IgG and nAb responses against ^RBD^MERS‐CoV after two doses and moderate responses ^omicron's‐RBD^SARS‐CoV‐2 after the third dose. Remarkably, it also generated strong cross‐reactive antibodies against SARS‐CoV‐1. These findings suggest that ^RBD^MERS‐CoV is more immunogenic than ^omicron's‐RBD^SARS‐CoV‐2, and that BiVax_Fc‐FcRn_ has a high potential for further development as a broad‐spectrum vaccine platform to prevent infection with the targeted coronaviruses as well as future emerging viruses.

## 1 Introduction

Coronaviruses (CoVs) are highly pathogenic viruses that infect various animal species and humans. Of the seven known human coronaviruses (HCoVs), MERS‐CoV and SARS‐CoV‐2 have caused major outbreaks over the past few years [1, 2]. While MERS‐CoV is associated with higher mortality rates, SARS‐CoV‐2 has demonstrated greater transmissibility, resulting in the COVID‐19 pandemic [3]. All of these viruses use the spike (S) protein to enter host cells, with the receptor‐binding domain (RBD) within the S1 subunit serving as the key region for binding [4]. Although vaccines targeting SARS‐CoV‐2 have been widely deployed, challenges such as waning immunity and reduced efficacy against emerging variants remain unresolved [5]. Moreover, no FDA‐approved vaccines exist for MERS‐CoV, leaving a crucial gap in pandemic preparedness [6].

Fc‐fusion vaccines have emerged as an alternative vaccination strategy, offering several advantages over conventional vaccines. These constructs fuse an antigen (such as a viral RBD) to the crystallizable fragment (Fc) region of IgG antibodies, thereby providing antibody‐like properties. The Fc domain can enhance vaccine immunogenicity through immune effector functions, dimerization for multivalency, extended serum half‐life via neonatal Fc receptor (FcRn) binding [7], and facilitates Fcγ receptor (FcγR)‐mediated antigen uptake by antigen‐presenting cells [8]. These properties lead to enhanced B‐cell activation and T‐cell priming, resulting in more potent and durable immune responses. Several studies have demonstrated the immunogenicity and efficacy of Fc‐fusion vaccines. For instance, SARS‐CoV‐2 RBD‐Fc constructs have been shown to induce higher titers of neutralizing antibodies (nAbs) than monomeric RBDs in non‐human primates and human studies [9].

**Figure 1:**
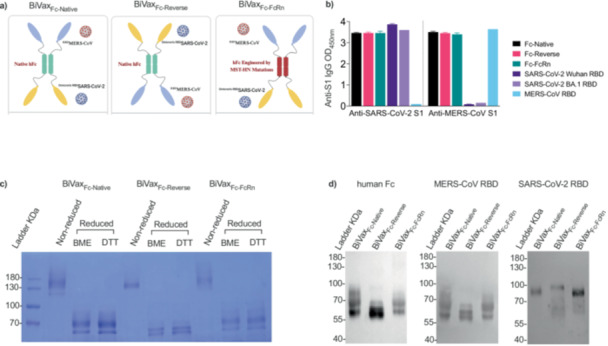
Design and characterization of BiVax vaccines. (a) Schematic representation of the three BiVax vaccines. Fc‐Native, Fc‐Reverse, and Fc‐FcRn. Each design incorporates the RBDs of MERS‐CoV and SARS‐CoV‐2 Omicron (BA.1) fused to the human IgG1 Fc region in varying orientations. (b) ELISA detection of RBD antigens demonstrates recognition of the BiVax constructs by polyclonal anti‐S1 antibodies, indicating antigenic integrity, expression, and antigenic accessibility of the MERS‐CoV RBD and SARS‐CoV‐2 RBD domains. (c) SDS‐PAGE gel showing purified BiVax vaccines (BiVax_Fc‐Native_, BiVax_Fc‐Reverse_, and BiVax_Fc‐FcRn_) under non‐reduced and reduced conditions (with BME or DTT) and stained with Coomassie Blue. (d) Western Blot analysis of the different parts of the three purified BiVax vaccines was performed using antibodies against Fc, MERS‐CoV RBD, and SARS‐CoV‐2 Omicron (BA.1) RBD.

In response to the urgent need for cross‐protective and more effective vaccines, we developed a multi‐target Fc‐based vaccine platform, termed BiVax, by engineering the Fc domain of human IgG1 to create three multivalent vaccines: BiVax_Fc‐Native_, BiVax_Fc‐Reverse_, and BiVax_Fc‐FcRn_ (Figure 1a). The BiVax_Fc‐Native_ and BiVax_Fc‐Reverse_ platforms feature a multivalent strategy wherein the RBDs of MERS‐CoV and SARS‐CoV‐2 Omicron are fused to human IgG Fc in opposite configurations to assess the impact of the RBDs location on the vaccine's immunogenicity. BiVax_Fc‐FcRn_ incorporates MST‐HN mutations [10], also known as ABDEG (M252Y/S254T/T256E‐H433K/N434F), which enhance binding to FcRn expressed in a wide variety of cells, including antigen‐presenting cells [7], to improve vaccine immunogenicity.

## 2 Materials and Methods

### 2.1 Vaccines Design and Synthesis

Three BiVax vaccines were designed by fusing the sequences of ^Omicron's‐RBD^SARS‐CoV‐2 (Sequence ID: WRW33943.1) to the hinge, CH2, and CH3 of human IgG1‐Fc (hFc) (Sequence ID: 7LBL_A) from one side and the ^RBD^MERS‐CoV (Sequence ID: AYN72346.1) from the other using Glycine‐Serine (G_4_S)_3_ linkers. The first candidate BiVax_Fc‐Native_ comprised a native hFc fused with ^RBD^MERS‐CoV at the N‐terminus and the ^Omicron's‐RBD^SARS‐CoV‐2 at the C‐terminus. The second vaccine BiVax_Fc‐Reverse_ also has a native Fc, but the ^RBD^MERS‐CoV is fused to the C‐terminus and the ^Omicron's‐RBD^SARS‐CoV‐2 to the N‐terminus. The third design, BiVax_Fc‐FcRn_, resembles the BiVax_Fc‐Native_ design, though the Fc domain incorporates the MST‐HN mutations (M252Y/S254T/T256E‐H433K/N434F). The gene sequences of the three vaccines were codon optimized for CHO cells expression, commercially synthesized (Genscript, Piscataway, NJ, USA), and cloned into the pcDNA 3.1 (+) mammalian expression vector (Invitrogen, Carlsbad, CA, USA). The DNA used for transfection was prepared using the GenElute HP Plasmid Maxiprep kit (Sigma‐Aldrich, Germany).

### 2.2 Proteins Expression and Purification

ExpiCHO‐S cells (Gibco Carlsbad, CA, USA) were cultured and transfected with BiVax_Fc‐Native_, BiVax_Fc‐Reverse_, and BiVax_Fc‐FcRn_ plasmids in a similar manner as previously described [11]. On day 8 post‐transfection, the supernatants of the expressed proteins were harvested by centrifugation and filtered using a 0.22 µm filter before purification using protein‐A chromatography. The HiTrap MabSelect SuRe (Cytiva, Marlborough, MA, USA) column was equilibrated with ×1 PBS prior to loading the filtered supernatant. The column was subsequently washed with ×1 PBS buffer. The recombinant vaccine was then eluted with 0.1 M glycine at pH 3 and neutralized with 1 M Tris HCl at pH 8 immediately, ensuring that the exposure to low pH was brief and mitigated by rapid pH correction. The proteins were desalted into PBS using a HiPrep 26/10 column (Cytiva, Marlborough, MA, USA) and filtered with a 0.22 µm filter. All proteins were kept on ice during purification and handled gently to minimize denaturation. The concentration of the eluted protein was determined at 280 nm using a Nanodrop 1000 spectrophotometer (Thermo Fisher Scientific, USA) and the calculated extinction coefficient.

### 2.3 SDS‐PAGE and Western Blotting

SDS‐PAGE was performed to determine the purity and molecular mass of the proteins of interest. In brief, 8% sodium dodecyl sulfate‐polyacrylamide (SDS) gel electrophoresis was used. SDS sample buffer (4×) alone was used as a loading buffer for non‐reducing conditions, or with 2‐mercaptoethanol (BME) or Dithiothreitol (DTT) as a denaturing buffer for reducing conditions. Samples were prepared to be 2 μg, and the samples containing the reducing agents were boiled at 100°C for 10 min, while the remaining samples were kept at room temperature. After loading the samples, the gel was run in a 1× running buffer for 10 min at 80 V, then increased to 100 V for 90 min. This was followed by Coomassie blue staining for 10 min at room temperature, and destaining twice for 15 min then overnight. A prestained protein ladder‐extra broad molecular weight (10–180 kDa) (Thermo Fisher Scientific, USA) was used to estimate the molecular mass of the purified proteins.

Western blotting was used to establish each vaccine fragment. Briefly, after SDS‐PAGE gel electrophoresis, proteins were transferred to a polyvinylidene fluoride (PVDF) membrane. After blocking with Tris‐buffered saline with 0.1% Tween (TBS‐T) and 5% skimmed milk, each vaccine component was detected using its primary antibody. The membranes were incubated at room temperature for 1 h with serum mouse primary polyclonal antibodies against SARS‐CoV‐2 S1 (in‐house) and MERS‐CoV (in‐house) S1 at a 1:1000 dilution in blocking buffer. To detect Fc, the membrane was incubated with Peroxidase AffiniPure Goat Anti‐Human IgG, Fcγ fragment‐specific antibody (from goat) (Jackson Immunoresearch, UK) at a 1:10,000 dilution in blocking buffer. After that, membranes were washed three times, 15 min each, with TBS‐T before incubating membrane for 1 h with HRP Goat anti‐mouse IgG (minimal x‐reactivity) antibody (Jackson ImmunoResearch, UK) at 1:5000 dilution for membranes detected SARS‐CoV‐2 and MERS‐CoV. The membranes were then washed three times. ECL Substrate was used (Bio‐Rad, USA) for reading. The iBright FL1500 (Invitrogen, Carlsbad, CA, USA) detection system was used for detection.

### 2.4 Detection of BiVax Fragments

The expression of the different BiVax vaccine RBDs fragments was determined using indirect ELISA. Briefly, 96‐well plates were coated with 1 μg/mL of recombinant MERS‐CoV RBD, SARS‐CoV‐2 RBD (Wuhan), or SARS‐CoV‐2 RBD Omicron (Sinobiological) and purified recombinant BiVax vaccines in PBS overnight at 4°C. Then, the plates were washed with PBS containing 0.05% Tween 20 (PBS‐T) and blocked with (300 μL/well) 5% skim milk in PBS‐T buffer (blocking buffer) for 2 h at room temperature. After washing, the plates were incubated with in‐house mouse sera against MERS‐CoV S1 and SARS‐CoV‐2 S1 diluted at 1:100 dilution in blocking buffer and incubated for 1 h at 37°C. After three washes, HRP Goat anti‐mouse IgG antibody (Jackson ImmunoResearch, UK) at 1:5000 dilution was added as a secondary antibody and incubated for 1 h at 37°C (100 μL/well). Excess secondary antibody was removed by three washes, and color was developed by adding 100 μL of 3,3′,5,5′‐tetramethylbenzidine (TMB) substrate (KPL, Gaithersburg, MD, USA) for 15 min. Finally, the reaction was stopped with 0.16 M sulfuric acid, and the absorbance was read spectrophotometrically at 450 nm using a Synergy 2 Multi‐Detection Microplate Reader (BioTek, Winooski, VT, USA).

### 2.5 Ethics Statement for Animal Study

Six to eight‐week‐old female BALB/c mice, which are frequently used to evaluate the efficacy of vaccine candidates [12, 13], were in good health. Animal studies were conducted in the King Fahad Medical Research Center, and the animal protocol was approved with the Animal Care and Use Committee's (ACUC) permission (ACUC‐20‐03‐9‐11).

### 2.6 Mice Immunization

Eight groups (*n* = 9) of 6‐ to 8‐week‐old female BALB/c mice, with weights ranging from 20 to 26 g were used in this study. The mice were housed in conditions with controlled room temperature and humidity, a 12:12 light:dark cycle, and unrestricted access to food and water. Mice received three doses of the different vaccines, with doses equal to 10 μg, intramuscularly (IM) or subcutaneously (SC). All vaccines were mixed with an equal volume of aluminum hydroxide gel (alum) adjuvant (ALHYDRGEL 2%, InvivoGen). Control groups received PBS plus adjuvant. The vaccines were administered 21 days apart. On days 0, 21, 42, and 63, blood samples were collected from the mice via retro‐orbital route.

### 2.7 Enzyme‐Linked Immunosorbent Assay (ELISA)

Total binding IgG was detected using indirect ELISA, as reported in a previous study [14]. Briefly, 96‐well plates were coated with 1 μg/mL of MERS‐CoV RBD, SARS‐CoV‐2 S1 (Wuhan), SARS‐CoV‐1 RBD, and SARS‐CoV‐2 RBD Omicron in PBS overnight at 4°C. Then, the plates were washed with PBS containing 0.05% Tween 20 (PBS‐T) and blocked with (300 μL/well) 5% skim milk in PBS‐T buffer (blocking buffer) for 2 h at room temperature. After washing, two‐fold serially diluted sera (100 μL/well) starting from a 1:100 dilution in blocking buffer were added and incubated for 1 h at 37°C. Some samples were only tested at a 1:100 dilution. After three washes, HRP Goat anti‐mouse IgG (minimal x‐reactivity) antibody at 1:5000 dilution (Jackson ImmunoResearch, UK) was added as secondary antibody and incubated for 1 h at 37°C (100 μL/well). Excess secondary antibody was removed by three washes, and color was developed by adding 100 μL of 3,3′,5,5′‐TMB substrate (KPL, Gaithersburg, MD, USA) for 15 min. Finally, the reaction was stopped with 0.16 M sulfuric acid, and the absorbance was read spectrophotometrically at 450 nm using a Synergy 2 Multi‐Detection Microplate Reader (BioTek, Winooski, VT). The endpoint titers were calculated by taking the reciprocal of the highest dilution that had an optical density (OD) value over 0.1, which was set as the threshold OD value.

### 2.8 Pseudovirus Microneutralization Assay

The evaluation of nAbs in vaccinated mice was performed under BSL‐2 conditions by using recombinant vesicular stomatitis virus (rVSV)‐based pseudoviruses expressing the SARS‐CoV‐2 of Wuhan strain, SARS‐ CoV‐2 of Omicron BA.1 strain, MERS‐CoV and SARS‐ CoV‐1 spike proteins, as previously described [15, 16]. Briefly, rVSV pseudoviruses were generated in Baby Hamster Kidney BHK21/WI‐2 cells (Kerafast, USA) transfected with plasmids expressing the corresponding proteins using lipofectamine 2000 (Invitrogen, Carlsbad, CA, USA). After 24 h of transfection, the cells were infected with rVSV‐ΔG/G*‐luciferase. The virus inoculum was removed after 1 h, the cells were washed twice with PB, and incubated in complete 5% DMEM containing rabbit polyclonal anti‐VSV‐G antibody at 1:1000, to remove any excess rVSV‐ΔG/G*‐luciferase for 24 h at 37°C and 5% CO_2_. The supernatant containing the generated pseudovirus was harvested 24 h post‐infection, and the virus titer was determined by measuring luciferase activity expressed as relative luciferase units (RLU) from two‐fold serially diluted pseudovirus on Vero E6 cells. The neutralization assay was performed by incubating equal volumes of two‐fold serially diluted heat‐inactivated sera, prepared in DMEM with 5% FBS, starting from a 1:20 dilution with DMEM containing 5 × 10^4^ RLU of each pseudovirus in duplicates for 1 h at 37°C in a 5% CO_2_ incubator. Then, 100 μL of the pseudovirus‐serum mixtures were transferred onto Vero E6 cell monolayers and incubated at 37°C in a 5% CO_2_ humidified incubator for 24 h. Cell control (CC) and virus control (VC) were included in each assay run. After 24 h of incubation, the media were removed, and the cells were washed with PBS, lysed with ×1 lysis buffer, and luciferase activity was measured by adding 50 μL luciferase substrate and 100 μL luciferase buffer to each well. After that, the luminescence activity was quantified using a BioTek Synergy 2 microplate reader (BioTek, Winooski, VT, USA). The 50% Inhibitory Concentration (IC_50_) neutralization titers were determined using a four‐parameter logistic (4PL) curve in GraphPad Prism V9 software (GraphPad Co., San Diego, CA, USA).

### 2.9 Statistical Analysis

Statistical parameters, including the mean, standard deviation (SD), *p*‐values, and types of statistical tests, are reported in the graphs and corresponding legends. GraphPad Prism software version V9 (GraphPad Co., San Diego, CA, USA) was used for data analysis and graphical presentations. Statistical significance for binding IgG responses and nAbs was evaluated using one‐way ANOVA with Tukey's post hoc test. Significance levels were defined as follows: *p < 0.05, **p < 0.01, ***p < 0.001, and ****p < 0.0001. Graphics and illustrations were created using BioRender and GraphPad Prism software version 10.1.1.

## 3 Results

### 3.1 Protein Expression and Characterization

The three vaccines were produced and purified using ExpiCHO‐S cells and Protein‐A affinity chromatography. Transfection produced an average of 5 mg of the recombinant protein from a 100 mL starter culture volume. ELISA analysis confirmed the presence of MERS‐CoV^RBD^ and SARS‐CoV‐2^RBD^ fragments across the three BiVax platforms (Figure 1b). SDS‐PAGE analysis under non‐reducing conditions revealed protein bands of ~150 kDa, indicating a dimer generated via disulfide bond due to the presence of the hFc domain. While under reducing conditions, the disulfide bonds were reduced to monomers (~70 kDa) (Figure 1c). Under reducing conditions, Western blot analysis revealed clear protein bands at approximately 70 kDa for all BiVax vaccine constructs (Fc‐Native, Fc‐Reverse, and Fc‐FcRn) when probed with antibodies specific for human Fc, MERS‐CoV^RBD^, and SARS‐CoV‐2^RBD^ (Figure 1d). These findings confirm the presence of monomeric Fc‐fusion proteins and validate the successful incorporation and antigenicity of each respective domain in the purified vaccine constructs.

### 3.2 Adjuvanted BiVax_Fc‐FcRn_ Induces Robust IgG Antibodies in BALB/c Mice via IM Administration

**Figure 2:**
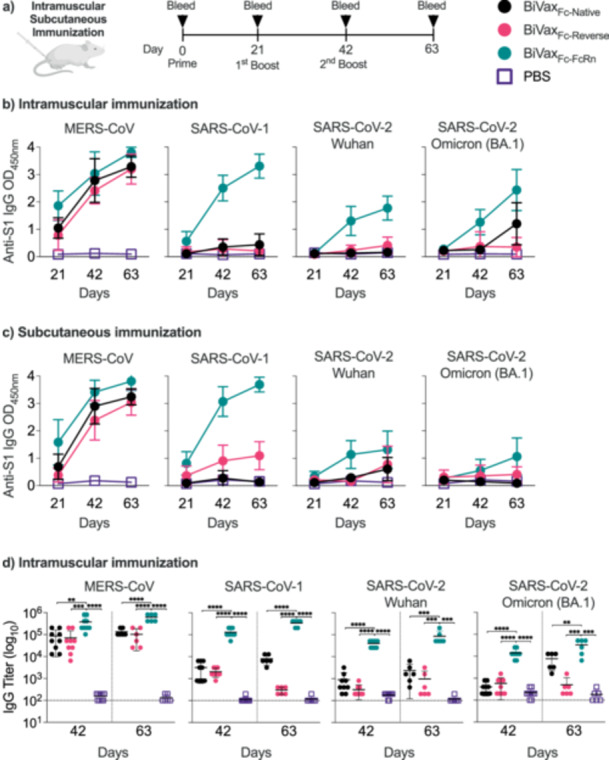
Binding IgG response. (a) Representative study design. Female BALB/c mice were randomly divided into 4 experimental groups (*n* = 9/group) and immunized intramuscularly or subcutaneously on days 0, 21, and 42 with one of three different vaccines, Fc‐Native, Fc‐Reverse, or Fc‐FcRn, or PBS. Blood samples were collected on days 0, 21, 42, and 63. Binding antibodies elicited by (b) intramuscular or (c) subcutaneous vaccinations. Mean optical density (OD) values of total S1‐specific binding IgG at 1:100 dilution from immunized mice were determined by ELISA on days 21, 42, and 63. (d) Endpoint titers of S1‐specific total binding IgG induced by intramuscular vaccination were determined by ELISA from immunized mice on days 42 and 63. S1 from MERS‐CoV, SARS‐CoV‐1, SARS‐CoV‐2 Wuhan, and SARS‐CoV‐2 Omicron (BA.1) were used in ELISA. Data are presented as mean ± SD. Statistical significance was determined by one‐way ANOVA analysis of variance with Tukey's post hoc test. Significance was reported as *p < 0.05, **p < 0.01, ***p < 0.001, and ****p < 0.0001. Dotted lines represent the cutoffs of the assays.

Mice were immunized via IM route on days 0, 21, and 42 with one of three different vaccines, BiVax_Fc‐FcRn_, BiVax_Fc‐Native,_ and BiVax_Fc‐Reverse_, or PBS. Blood samples were collected on days 0, 21, 42, and 63 to assess IgG antibody responses (Figure 2a). Immunization with the BiVax_Fc‐FcRn_ construct resulted in a robust and constant IgG response against MERS‐CoV and Omicron BA.1, although the response to Omicron BA.1 was lower. As shown in (Figure 2b), RBD‐specific binding antibodies were significantly elevated by day 42 and reached peak levels by day 63 across all tested viral antigens. Despite not encoding SARS‐CoV‐2 Wuhan or SARS‐CoV‐1 RBDs in the vaccines, BiVax_Fc‐FcRn_ induced measurable cross‐reactive IgG responses against these two viruses. This cross‐reactive response suggests the presence of shared epitopes or structural homology between the RBD domains, which were effectively presented by the BiVax_Fc‐FcRn_ platform. These findings demonstrate that stronger binding to FcRn can promote a broad and durable humoral immune response following intramuscular delivery.

In contrast to BiVax_Fc‐FcRn_, both BiVax_Fc‐Native_ and BiVax_Fc‐Reverse_ demonstrated reduced immunogenicity, and the IgG levels induced by these two constructs were minimal and largely restricted to MERS‐CoV, with no significant cross‐reactivity observed against SARS‐CoV‐2 Wuhan or SARS‐CoV‐1. While BiVax_Fc‐Native_ generated a weak but detectable IgG response to Omicron BA.1 on day 63, the magnitude was substantially lower than that induced by BiVax_Fc‐FcRn_. BiVax_Fc‐Reverse_ exhibited the lowest overall response levels, with no significant difference from the PBS control group across most antigens. These observations suggest that neither the unmodified Fc nor the reverse‐oriented fusion design is as efficient in driving cross‐reactive humoral responses in this bivalent vaccine model as BiVax_Fc‐FcRn_. Collectively, these results underscore the critical role of FcRn binding in enhancing antigen presentation and immunogenicity within the Fc‐fusion vaccine platform.

### 3.3 BiVax Vaccines Elicit IgG Response via SC Administration

**Figure 3:**
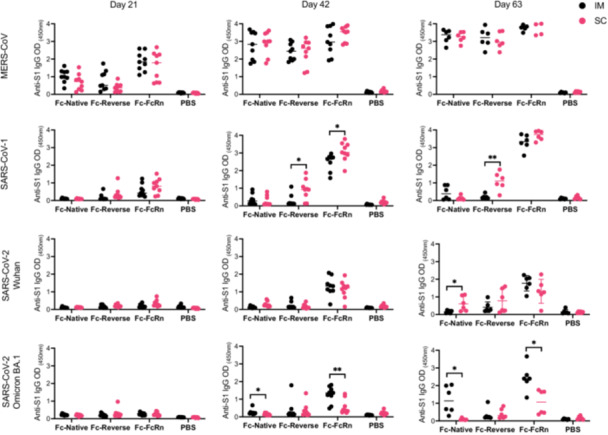
Comparison of intramuscular and subcutaneous IgG responses induced by BiVax vaccines. Binding IgG responses against RBD proteins from MERS‐CoV, SARS‐CoV‐1, SARS‐CoV‐2 Wuhan, and SARS‐CoV‐2 Omicron BA.1 measured by ELISA in mouse sera collected on days 21, 42, and 63 after intramuscular (IM, black) or subcutaneous (SC, pink) administration of BiVax_Fc‐Native_, BiVax_Fc‐Reverse_, or BiVax_Fc‐FcRn_. Each dot represents an individual mouse, and the values represent the anti‐RBD IgG optical density (OD) at 450 nm at a 1:100 serum dilution. Statistical analysis was performed using Mann–Whitney test; *p < 0.05, **p < 0.01.

The SC route generated detectable antigen‐specific responses, particularly against MERS‐CoV and SARS‐CoV‐1, as observed on days 42 and 63 (Figures 2c and 3). The trend of IgG responses mirrored those from IM immunization, but the magnitude was reduced against SARS‐CoV‐2 Wuhan and Omicron BA.1 S1 proteins (Figure 3). Despite this variability, BiVax_Fc‐FcRn_ elicited IgG responses against MERS‐CoV and SARS‐CoV‐1, with comparatively lower responses observed against SARS‐CoV‐2 Wuhan and Omicron BA.1. These findings suggest that while SC delivery can elicit target‐specific and cross‐reactive IgG responses, IM administration remains more consistent and effective in generating robust and homogeneous antibody responses, particularly against SARS‐CoV‐2 (Figures 2b,c and 3).

### 3.4 Intramuscular Immunization With BiVax_Fc‐FcRn_ Induces High Titers of Cross‐Reactive IgG Antibodies Against Homologous and Heterologous Coronavirus Antigens

To assess the breadth of the humoral immune response, endpoint ELISA was performed on sera collected from mice immunized IM with BiVax vaccine constructs or PBS as a control. IgG binding responses were evaluated on days 42 and 63 against spike RBD antigens from MERS‐CoV, SARS‐CoV‐1, SARS‐CoV‐2 Wuhan, and SARS‐CoV‐2 Omicron BA.1 (Figure 2d). Among all the groups, BiVax_Fc‐FcRn_ induced the highest IgG titers. Specifically, on day 63, IgG responses against the homologous antigens MERS‐CoV and Omicron BA.1 were significantly elevated compared to all other groups, with titers reaching 10^5^−10^6^. Notably, the response to Omicron was markedly enhanced after the third dose, as seen on day 63, indicating a strong booster effect. Furthermore, BiVax_Fc‐FcRn_ elicited statistically significant cross‐reactive IgG responses against SARS‐CoV‐1 and Wuhan, despite these antigens not being encoded in the vaccine. These responses were also significantly higher than those in the BiVax_Fc‐Native_, BiVax_Fc‐Reverse_, and PBS groups, demonstrating meaningful cross‐reactivity. In contrast, BiVax_Fc‐Native_ and BiVax_Fc‐Reverse_ induced only limited responses, mainly toward MERS‐CoV, and showed no statistically significant reactivity to heterologous antigens. No responses were detected in the PBS controls. Overall, these findings suggest that BiVax_Fc‐FcRn_ is significantly more immunogenic than the other BiVax constructs when delivered IM, both in terms of magnitude and breadth of the IgG response.

### 3.5 BiVax Constructs Strongly Neutralize MERS‐CoV and SARS‐CoV‐1 But Show Limited Activity Against Ancestral SARS‐CoV‐2 Wuhan Strain and Omicron BA.1 Variant

**Figure 4:**
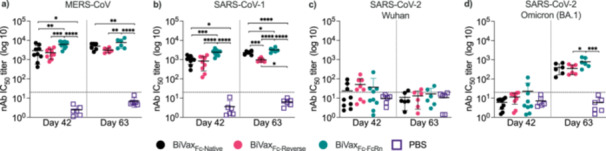
The median inhibitory concentration (IC_50_) of neutralizing antibodies (nAbs). Serum samples collected from immunized mice were tested for nAbs. The IC_50_ of nAbs in sera obtained on days 42 and 63 from mice immunized with the BiVax vaccines or PBS group were determined against rVSV‐ΔG‐based pseudoviruses expressing spike proteins from (a) MERS‐CoV, (b) SARS‐CoV‐1, and (c) ancestral Wuhan and (d) Omicron (BA.1) strains of SARS‐CoV‐2. Data are presented as mean ± SD. Statistical significance was determined by one‐way ANOVA analysis of variance with Tukey's post hoc test. Significance was reported as *p < 0.05, **p < 0.01, ***p < 0.001, and ****p < 0.0001. Dotted lines represent the cutoffs of the assays.

The neutralizing capacity of antibodies induced by the BiVax vaccines was evaluated against MERS‐CoV, SARS‐CoV‐1, SARS‐CoV‐2 (Wuhan), and SARS‐CoV‐2 (Omicron BA.1) pseudoviruses using sera from days 42 and 63. For MERS‐CoV (Figure 4a), the IC_50_ titers were 2.9 × 10^3^, 2.3 × 10^3^, 6.2 × 10^3^ on day 42 and boosted to 4.9 × 10^3^, 3.1 × 10^3^, and 7.7 × 10^3^ on day 63 for BiVax_Fc‐Native_, BiVax_Fc‐Reverse_, and BiVax_Fc‐FcRn_, respectively. Interestingly, BiVax_Fc‐Native_ and BiVax_Fc‐Reverse_ elicited nAbs with IC_50_ titers of 1.0 × 10^3^ and 0.8 × 10^3^ on day 42 and titers of 2.3 × 10^3^ and 1.0 × 10^3^ on day 63 against SARS‐CoV‐1 (Figure 4b). These nAbs were observed despite negative results for binding antibodies (Figure 2b), indicating that the induction of nAbs is not necessarily associated with the presence of binding antibodies. Importantly, vaccination with BiVax_Fc‐FcRn_ resulted in significantly higher SARS‐CoV‐1 nAb titers (2.3 × 10^3^ boosted to 3.2 × 10^3^ after the third dose) than those with BiVax_Fc‐Native_ and BiVax_Fc‐Reverse_ formats. Following the third dose, all immunized mice exhibited more consistent nAb responses, with reduced intra‐group variability compared to earlier time points, indicating more synchronized and focused immune activation (Figure 4a,b). Collectively, these findings indicate that the BiVax platform, regardless of Fc modification, is broadly effective in generating cross‐neutralizing immunity against MERS‐CoV and SARS‐CoV‐1, with BiVax_Fc‐FcRn_ demonstrating the highest level of immunogenicity.

In contrast, all BiVax vaccines elicited only limited nAbs against the ancestral SARS‐CoV‐2 Wuhan strain, even after three doses, reflecting the antigenic divergence between MERS‐CoV and SARS‐CoV‐2 RBDs (Figure 4c) and consistent with the observed weak binding IgG levels. Similarly, nAb levels against SARS‐CoV‐2 Omicron BA.1 were low after two doses on day 42 but increased after the third dose to 3.0 × 10^2^ in the BiVax_Fc‐Native_ and BiVax_Fc‐Reverse_ groups (Figure 4d). On the other hand, BiVax_Fc‐FcRn_ outperformed the other formats by eliciting significantly higher levels of nAbs, reaching 7.8 × 10^2^ on day 63, further confirming its superior immunogenicity.

Overall, BiVax_Fc‐FcRn_ induced the highest nAb titers against homologous antigens (MERS‐CoV and Omicron BA.1) as well as the heterologous SARS‐CoV‐1 among all tested coronavirus antigens.

## 4 Discussion

Understanding the structural and immunological behavior of recombinant subunit vaccines is critical to advance vaccine platforms against high‐risk CoVs and other pathogens. We generated a panel of Fc‐based BiVax constructs with MERS‐CoV and SARS‐CoV‐2 Omicron BA.1 RBDs, and evaluated their expression, antigen integrity, and ability to elicit cross‐reactive and functional humoral immunity in a murine model. Comparisons of the delivery route and boosting impact further provided insights into how our design and immunization strategy affected the overall vaccine efficacy.

The expected bands were observed for all constructs in SDS‐PAGE under non‐reducing conditions, near the expected size of ~150 kDa. Under reducing conditions, the multiple observed bands primarily corresponded to the constituent RBD‐Fc polypeptides and potential breakdown products, rather than unrelated contaminants. Although no further polishing steps were performed, the high purity of the preparations was consistent with the strong and specific neutralizing responses observed in vivo. Additionally, Western blot analysis showed that polyclonal antibodies generated against the S1 protein from either SARS‐CoV‐2 or MERS‐CoV readily bound to the vaccines. Furthermore, ELISA confirmed the presence of MERS‐CoV^RBD^ and SARS‐CoV‐2^RBD^ fragments within the BiVax constructs, indicating that the antigens were expressed and presented in a conformationally compatible format for antibody recognition, demonstrating that immunologically relevant epitopes are accessible on the purified proteins (Figure 1b). However, it should be noted that polyclonal antibodies recognize both linear and conformational determinants; and therefore, binding by such sera cannot be considered a strict proxy for or definitive proof of correct native‐state folding. A more rigorous assessment would require conformationally specific anti‐RBD monoclonal antibodies that recognize defined structural epitopes or direct biophysical methods such as surface plasmon resonance (SPR), biolayer interferometry (BLI), or differential scanning fluorimetry (DSF), which should be included in the future characterization of the BiVax platform.

Nonetheless, the appearance of multiple bands in the SDS‐PAGE and Western blot under reducing conditions, especially in the Fc and MERS detectors, can be attributed to several well‐documented factors associated with IgG‐based multispecific constructs expressed in ExpiCHO cells. First, SDS‐PAGE sample preparation itself may introduce artifacts, including partial dissociation or disulfide scrambling, which can generate additional bands without representing true molecular heterogeneity [17]. Second, the ExpiCHO expression system, which are known for their very high productivity, has been reported to increase the likelihood of producing truncated or partially assembled species, particularly in structurally complex multispecific formats. The high protein synthesis rate in ExpiCHO cells can impose folding stress or lead to incomplete translation, resulting in lower‐molecular‐weight products that are detectable by Western blot [18, 19]. Furthermore, it has been proposed that the high translation rate in ExpiCHO‐S cells could reduce folding accuracy, while N‐ethylmaleimide (NEM) was shown to suppress the formation of such artifacts [18]. Thus, it is essential, in future stages of advancing this platform, to conduct expanded analytical assessments, including mass spectrometry, aggregation profiling, and stability testing, to establish a robust product‐quality profile that will guide bioprocess optimization and early formulation development [20]. In this context, it is important to clarify that the purification using HiPrep 26/10 desalting column used in our downstream processing is a buffer‐exchange step designed to remove small‐molecule impurities below approximately 5 kDa; it does not resolve monomeric protein from high‐molecular‐weight aggregates, which could co‐elute in the void volume of the column. Accordingly, the presence of soluble oligomeric or aggregated species in the BiVax preparations cannot be excluded based solely on the desalting step. Analytical size‐exclusion chromatography (SEC), using columns such as Superdex 200 or Superdex 300, is the appropriate method for assessing the native aggregation state and apparent molecular weight of Fc‐fusion proteins in solution. It should be incorporated as a standard quality attribute in future studies and any regulatory‐facing analytical characterization package for the BiVax platform. However, as seen in the SDS‐PAGE gel (Figure 1c), no material was observed in the wells, suggesting that macro‐aggregation was not significant in these preparations, although definitive confirmation of the absence of soluble oligomeric aggregates requires further analytical testing. Furthermore, the use of acidic buffers for elution from Protein A is a well‐established method for purifying conventional IgG1‐based fusion proteins. However, the structural complexity and modularity of multispecific formats, such as the BiVax platform, may introduce additional developability considerations, including higher susceptibility to aggregation, fragmentation, or conformational instability during upstream and downstream bioprocesses [21]. Therefore, further enhancements of the BiVax platform require exploring other purification strategies, and linker or molecular design elements that could mitigate potential misfolding and support the developability of multispecific Fc‐fusion vaccines.

From an immunogenicity standpoint, all BiVax constructs successfully elicited moderate to strong IgG responses, particularly through the IM route. Among them, BiVax_Fc‐FcRn_ was the most effective in inducing binding and nAbs against a broad panel of CoVs antigens, including MERS‐CoV, SARS‐CoV‐1, SARS‐CoV‐2 Omicron, and, to a lesser extent, the ancestral SARS‐CoV‐2 Wuhan strain. The route and number of doses notably influenced the magnitude of the observed responses. However, SC administration was associated with a weaker immune response compared to the IM route against SARS‐CoV‐2 Wuhan and Omicron BA.1. This variability could be attributable to the pharmacokinetic and physiological limitations of SC delivery. Bioavailability after SC injection is influenced by several factors, including tissue structure, lymphatic drainage, and molecular size of biotherapeutics [22]. Given the high molecular weight of monoclonal antibodies (~150 kDa), their absorption via the SC route largely depends on the lymphatic system, which may result in delayed or reduced systemic exposure [23]. These host‐ and molecule‐specific barriers may help explain the higher consistency and potency seen with IM delivery.

The BiVax Fc‐based vaccines presented here are subunit formulations, known for their safety and tolerability [6]. The spike S1 RBD is essential for viral entry but is inherently small and weakly immunogenic, necessitating adjuvant use [24]. In this design, the Fc‐FcRn construct incorporated MST‐HN mutations to enhance the affinity for the neonatal FcRn, improving antigen presentation and extending the serum half‐life [25]. The BiVax_Fc‐FcRn_ construct fused MERS‐CoV and Omicron BA.1 RBDs to the human IgG Fc, resulting in robust immune responses in mice. Remarkably, although no SARS‐CoV‐1 RBD was present, Fc‐FcRn still induced cross‐reactive binding antibodies against SARS‐CoV‐1, similar to the findings by Cohen et al. [26].

BiVax_Fc‐Native_ elicited antibodies against the MERS‐CoV RBD but not the Omicron RBD. This may relate to the positioning of the RBD near the Fc hinge, promoting better exposure of MERS‐RBD. Alternatively, the immunogenicity of MERS‐RBD itself may be inherently stronger, as suggested by Da et al. [24]. To test this hypothesis, BiVax_Fc‐Reverse_ was designed to invert the orientation of the RBDs. This allowed for the evaluation of whether proximity to the hinge or intrinsic immunogenicity drove the observed differences. The results from the BiVax_Fc‐Reverse_ construct further supported the dominant immunogenicity of MERS‐RBD.

The finding that MERS‐CoV RBD consistently elicited stronger immune responses than Omicron BA.1 RBD, regardless of orientation within the bivalent construct, is of interest. While this may reflect a feature of multivalent vaccines in which one component (MERS‐CoV RBD in our case) is more immunodominant than the other, this observation is consistent with growing evidence that the Omicron BA.1 spike and RBD are intrinsically weak immunogens. Several studies have shown that BA.1 displays poor immunogenicity and elicits weak antibody responses in both humans and animal models, whether after infection or vaccination. This is largely attributed to the extensive mutations present in BA.1, particularly in the RBD, which severely alters the antigenic landscape and reduces antibody recognition.

It has been shown that primary infection with Omicron BA.1 in children resulted in low levels of antibody and neutralization activity [27, 28]. In addition, antibody titers in individuals with BA.1 breakthrough infections were substantially lower than those following Delta infection [29]. Similarly, vaccines based on Omicron BA.1 induced weaker antibody responses and poor neutralization compared to vaccines targeting the ancestral Wuhan strain or later variants [30]. Experimental models have also confirmed this observation, in which primary BA.1 infection in mice induced weak homologous nAbs with little boosting upon re‐exposure, mirroring the impaired immunogenicity seen with BA.1‐based vaccines [31]. This could be attributed to the unique antigenic features of BA.1 and its numerous RBD mutations. In fact, the BA.1 spike protein contains > 30 substitutions, insertions, or deletions, including at least 15 changes in the RBD, many of which localize to key antigenic sites and significantly impair antibody binding and neutralization [30]. These findings align with our observation that a good Omicron neutralizing antibody response was only achieved after a third immunization. Given the intrinsic limitations of BA.1‐based immunogens, future vaccine designs may benefit from incorporating updated RBDs derived from more immunogenic and antigenically relevant variants, as well as broader antigen screening, to enhance their cross‐protective potential.

The BiVax constructs demonstrated potent neutralizing activity against MERS‐CoV, supporting previous evidence of MERS antigen immunogenicity across vaccine platforms [24, 32]. The induction of cross‐neutralizing nAbs by BiVax vaccines against SARS‐CoV‐1 is intriguing and requires further investigation. Previous data indicate that antibodies induced by the SARS‐CoV‐1 RBD lack cross‐reactivity or cross‐neutralization against MERS‐CoV [33]. These observations suggest that while MERS‐CoV RBD might share some structural similarities with homologous domains of SARS‐CoV‐1, the antibodies targeting the SARS‐CoV‐1 RBD do not effectively recognize or neutralize MERS‐CoV. This lack of cross‐reactivity could be attributed to the distinctive utilization of receptors by the two CoVs, as MERS‐CoV uses DPP4 as its receptor, and SARS‐CoV‐1 uses ACE2. Despite the similarities in the core regions of the RBDs of SARS‐CoV‐1 and MERS‐CoV, the receptor‐binding motifs (RBMs) exhibit significant differences. These divergences in the RBM regions could contribute to their distinct recognition patterns toward different receptors [34]. Cross‐reactivity has instead been observed from SARS‐CoV‐2 infected but not vaccinated individuals, mainly against the conserved S2 subunit of MERS‐CoV and MERS‐related CoVs [35, 36]. Such cross‐reactivity was also shown by a structure‐guided stem‐based MERS‐CoV vaccine, which induced cross‐reactive antibodies against the conserved S2 region of both MERS‐CoV and SARS‐CoV‐2 [37].

The high sequence similarity of 71.30% between Omicron RBD and SARS‐CoV‐1 RBD, compared to only 23.93% similarity with MERS‐CoV RBD, suggests a possible cross‐reactivity. However, the weak magnitude of the immune response elicited against SARS‐CoV‐2 Wuhan or Omicron BA.1 by the BiVax vaccines does not reflect the potency of the cross‐reactivity against SARS‐CoV‐1. Thus, we believe that cross‐reactivity against SARS‐CoV‐1 is from MERS‐CoV RBD rather than SARS‐CoV‐2 Omicron RBD in our vaccine formulation, as evidenced by cross‐binding [38, 39] and cross‐neutralization (unpublished data) of SARS‐CoV‐1 by sera from acutely infected MERS patients.

Importantly, the increase in neutralizing activity suggests that the administration of BiVax vaccines as a third dose can enhance the ability of the immune system to neutralize SARS‐CoV‐2 Omicron, thereby improving the overall efficacy of the vaccine in providing protection against this highly transmissible variant. In addition, Lusvarghi's work supports the crucial role of administering a third dose in producing nAbs that specifically target SARS‐CoV‐2 Omicron [40].

It is noteworthy that although binding IgG antibodies against SARS‐CoV‐2 Wuhan and Omicron were detected after vaccination, especially with the BiVax_Fc‐FcRn_ vaccine, neutralizing activity was initially limited. This discrepancy likely reflects the presence of non‐neutralizing or low‐avidity antibodies at earlier time points. A booster dose enhanced neutralization against Omicron but not Wuhan, likely due to the antigenic mismatch between the Omicron‐based immunogen and the Wuhan strain. This highlights that ELISA binding and neutralization do not always correlate with each other. Additionally, it is important to note that pseudovirus assays were used here, which correlate well with live virus neutralization assays, but future work should include validation with live SARS‐CoV‐2 strains. A significant limitation of this proof‐of‐concept study is the absence of a head‐to‐head comparison with a standard‐of‐care benchmark vaccine formulation or monovalent RBD controls. Without such a comparator, such as a recombinant spike protein, monovalent RBD with alum adjuvant, or an approved COVID‐19 vaccine, the absolute neutralization titers and binding antibody levels reported here cannot be fully contextualized in terms of relative potency, clinical relevance, or protective threshold. Our focus in this study was on the internal comparison of the three BiVax construct configurations against each other, and no external positive control was included. Nonetheless, the binding and nAbs against MERS‐CoV, for example, were in the range of 10^5^−10^6^ and ~10^3^, respectively, which are comparable to titers we have previously observed when immunizing mice with recombinant MERS‐CoV S1 protein adjuvanted with alum [41], suggesting that the BiVax platform generates antibody responses of biologically relevant magnitude against MERS‐CoV. Although direct cross‐study comparisons are constrained by differences in immunization schedules, antigen doses, and assay formats, this informal benchmark supports the conclusion that BiVax‐induced MERS‐CoV immunity is immunologically significant. Future studies must incorporate monovalent RBD‐alum and approved spike‐based vaccines as direct within‐experiment comparators to rigorously establish the relative immunogenicity and protective potential of the BiVax platform.

The use of human IgG1 Fc in a murine model could elicit anti‐Fc responses due to species mismatch; however, our assays specifically measured antibodies targeting coronavirus RBDs, not Fc proteins. While human Fc may provide an additional adjuvant effect in mice, it is considered non‐immunogenic in humans and has a strong safety profile, as evidenced by its widespread use in therapeutic antibodies. We selected IgG1 for its potent effector functions, though future studies may explore alternative Fc isotypes, such as IgG4, to modulate immunogenicity or inflammatory responses.

BiVax_Fc‐FcRn_ produced the highest levels of nAbs against homologous antigens (MERS‐CoV and Omicron BA.1) and heterologous SARS‐CoV‐1. This response was significant only after booster administration, with a marked improvement in uniformity across individuals, indicating more synchronized immune activation. These results support the importance of a third dose in enhancing the functional antibody response, especially for immune‐evasive variants such as Omicron BA.1.

Using Fc‐based vaccines is a promising next‐generation vaccine strategy because of their compatibility with the production of recombinant proteins and the proven effectiveness in preclinical models [42]. Additionally, they can be leveraged to enhance immunogenicity through dimerization and FcRn engagement, improve pharmacokinetics via FcRn binding, and achieve multivalent targeting. This platform can be produced using the current commercial antibody manufacturing infrastructure as cost‐effective production at an industrial scale. These characteristics together suggest that the BiVax platform is a promising candidate for further development into a universal coronavirus and other pathogens.

## Author Contributions

M.A.A. and A.M.H. designed and conceptualized the work. A.H., N.D.A., A.G., R.M.A., F.A., A.A., M.A., A.Z., A.A.A., A.Almehmadi, J.B., T.A., and M.Y.K. performed and optimized the experiments and acquired the data. W.H.A., R.Y.A., A. Alnami, T.N., Turki S. Abujamel, M.A.A., and A.M.H. supervised the work. A.H., T.A., R.Y.A., M.A.A., and A.M.H. analyzed the data. A.H., T.A., Turki S. Abujamel, M.A.A., and A.M.H. drafted the manuscript. R.Y.A., A.Z., Turki S. Abujamel, M.A.A., and A.M.H. acquired funding. All authors have reviewed and edited the manuscript and agreed to the published version of the manuscript.

## Conflicts of Interest

M.A.A. and A.M.H. are inventors on patent applications related to this work. The remaining authors declare no conflicts of interest.

## Data Availability

The data that support the findings of this study are available from the corresponding author upon reasonable request.

## References

[1] S. P. Kenney , Q. Wang , A. Vlasova , K. Jung , and L. Saif , “Naturally Occurring Animal Coronaviruses as Models for Studying Highly Pathogenic Human Coronaviral Disease,” Veterinary Pathology 58, no. 3 (2021): 438–452, 10.1177/0300985820980842. 33357102

[2] World Health Organization , “Middle East Respiratory Syndrome Coronavirus (MERS‐CoV),” 2025, https://www.emro.who.int/health-topics/mers-cov/mers-outbreaks.html.

[3] A. R. Fehr and S. Perlman , “Coronaviruses: An Overview of Their Replication and Pathogenesis,” Methods in Molecular Biology 1282 (2015): 1–23, 10.1007/978-1-4939-2438-7_1. 25720466 PMC4369385

[4] M. Y. Wang , R. Zhao , L. J. Gao , X. F. Gao , D. P. Wang , and J. M. Cao , “SARS‐CoV‐2: Structure, Biology, and Structure‐Based Therapeutics Development,” Frontiers in Cellular and Infection Microbiology 10 (2020): 587269, 10.3389/fcimb.2020.587269. 33324574 PMC7723891

[5] A. Saha , S. Ghosh Roy , R. Dwivedi , et al., “Beyond the Pandemic Era: Recent Advances and Efficacy of SARS‐CoV‐2 Vaccines Against Emerging Variants of Concern,” Vaccines 13, no. 4 (2025): 424, 10.3390/vaccines13040424. 40333293 PMC12031379

[6] Y. D. Li , W. Y. Chi , J. H. Su , L. Ferrall , C. F. Hung , and T. C. Wu , “Coronavirus Vaccine Development: From SARS and MERS to COVID‐19,” Journal of Biomedical Science 27, no. 1 (2020): 104, 10.1186/s12929-020-00695-2. 33341119 PMC7749790

[7] K. Baker , T. Rath , M. Pyzik , and R. S. Blumberg , “The Role of FcRn in Antigen Presentation,” Frontiers in Immunology 5 (2014): 408, 10.3389/fimmu.2014.00408. 25221553 PMC4145246

[8] E. Richel , J. T. Wagner , S. Klessing , R. Di Vincenzo , V. Temchura , and K. Überla , “Antigen‐Dependent Modulation of Immune Responses to Antigen‐Fc Fusion Proteins by Fc‐Effector Functions,” Frontiers in Immunology 14 (2023): 1275193, 10.3389/fimmu.2023.1275193. 37868961 PMC10585040

[9] Z. Liu , W. Xu , S. Xia , et al., “RBD‐Fc‐Based COVID‐19 Vaccine Candidate Induces Highly Potent SARS‐CoV‐2 Neutralizing Antibody Response,” Signal Transduction and Targeted Therapy 5, no. 1 (2020): 282, 10.1038/s41392-020-00402-5. 33247109 PMC7691975

[10] P. Ulrichts , A. Guglietta , T. Dreier , et al., “Neonatal Fc Receptor Antagonist Efgartigimod Safely and Sustainably Reduces IgGs in Humans,” Journal of Clinical Investigation 128, no. 10 (2018): 4372–4386, 10.1172/JCI97911. 30040076 PMC6159959

[11] M. A. Alfaleh , R. M. Alsulaiman , S. A. Almahboub , et al., “ACE2‐Fc and DPP4‐Fc Decoy Receptors Against SARS‐CoV‐2 and MERS‐CoV Variants: A Quick Therapeutic Option for Current and Future Coronaviruses Outbreaks,” Antibody Therapeutics 7, no. 1 (2024): 53–66, 10.1093/abt/tbad030. 38371953 PMC10873275

[12] H. Gu , Q. Chen , G. Yang , et al., “Adaptation of SARS‐CoV‐2 in BALB/c Mice for Testing Vaccine Efficacy,” Science 369, no. 6511 (2020): 1603–1607, 10.1126/science.abc4730. 32732280 PMC7574913

[13] A. S. Cockrell , B. L. Yount , T. Scobey , et al., “A Mouse Model for MERS Coronavirus‐Induced Acute Respiratory Distress Syndrome,” Nature Microbiology 2, no. 2 (2016): 16226, 10.1038/nmicrobiol.2016.226. PMC5578707 27892925

[14] N. D. Aljehani , L. Tamming , M. Y. Khan , et al., “Mucosal SARS‐CoV‐2 S1 Adenovirus‐Based Vaccine Elicits Robust Systemic and Mucosal Immunity and Protects Against Disease in Animals,” mBio 16, no. 1 (2025): e0217024, 10.1128/mbio.02170-24. 39629990 PMC11708039

[15] S. A. Almahboub , A. Algaissi , M. A. Alfaleh , M. Z. ElAssouli , and A. M. Hashem , “Evaluation of Neutralizing Antibodies Against Highly Pathogenic Coronaviruses: A Detailed Protocol for a Rapid Evaluation of Neutralizing Antibodies Using Vesicular Stomatitis Virus Pseudovirus‐Based Assay,” Frontiers in Microbiology 11 (2020): 2020, 10.3389/fmicb.2020.02020. 33013745 PMC7498578

[16] T. A. Altorki , R. H. Abdulal , B. A. Suliman , et al., “Robust Memory Humoral Immune Response to SARS‐CoV‐2 in the Tonsils of Adults and Children,” Frontiers in Immunology 14 (2023): 1291534, 10.3389/fimmu.2023.1291534. 38149243 PMC10750384

[17] Z. C. Zhu , Y. Chen , M. S. Ackerman , et al., “Investigation of Monoclonal Antibody Fragmentation Artifacts in Non‐Reducing SDS‐PAGE,” Journal of Pharmaceutical and Biomedical Analysis 83 (2013): 89–95, 10.1016/j.jpba.2013.04.030. 23708435

[18] S. Hörner , M. Ghosh , J. Kauer , et al., “Mass Spectrometry for Quality Control of Bispecific Antibodies After SDS‐PAGE In‐Gel Digestion,” Biotechnology and Bioengineering 118, no. 8 (2021): 3069–3075, 10.1002/bit.27817. 33988851

[19] N. K. Jain , S. Barkowski‐Clark , R. Altman , et al., “A High Density CHO‐S Transient Transfection System: Comparison of ExpiCHO and Expi293,” Protein Expression and Purification 134 (2017): 38–46, 10.1016/j.pep.2017.03.018. 28342833

[20] K. Sampathkumar and B. A. Kerwin , “Roadmap for Drug Product Development and Manufacturing of Biologics,” Journal of Pharmaceutical Sciences 113, no. 2 (2024): 314–331, 10.1016/j.xphs.2023.11.004. 37944666

[21] V. Siegmund , D. Klewinghaus , J. Teroerde , et al., “Optimizing Colloidal Stability and Viscosity of Multispecific Antibodies at the Drug Discovery‐Development Interface: A Systematic Predictive Case Study,” mAbs 17, no. 1 (2025): 2553622, 10.1080/19420862.2025.2553622. 40888155 PMC12407640

[22] W. F. Richter and B. Jacobsen , “Subcutaneous Absorption of Biotherapeutics: Knowns and Unknowns,” Drug Metabolism and Disposition 42, no. 11 (2014): 1881–1889, 10.1124/dmd.114.059238. 25100673

[23] A. Datta‐Mannan , S. Estwick , C. Zhou , et al., “Influence of Physiochemical Properties on the Subcutaneous Absorption and Bioavailability of Monoclonal Antibodies,” mAbs 12, no. 1 (2020): 1770028, 10.1080/19420862.2020.1770028. 32486889 PMC7531508

[24] L. Dai , T. Zheng , K. Xu , et al., “A Universal Design of Betacoronavirus Vaccines Against COVID‐19, MERS, and SARS,” Cell 182, no. 3 (2020): 722–733.e11, 10.1016/j.cell.2020.06.035. 32645327 PMC7321023

[25] E. S. Ward , “IgG1 Antibodies With Mutated Fc Portion for Increased Binding to FcRn Receptor and Uses Thereof, EP1896503A2 (European Patent Office), E.E.P. Office),” 2023, https://patents.google.com/patent/EP1896503A2/en.

[26] A. A. Cohen , N. van Doremalen, , A. J. Greaney , et al., “Mosaic RBD Nanoparticles Protect Against Challenge by Diverse Sarbecoviruses in Animal Models,” Science 377, no. 6606 (2022): eabq0839, 10.1126/science.abq0839. 35857620 PMC9273039

[27] Z. Q. Toh , N. Mazarakis , J. Nguyen , et al., “Comparison of Antibody Responses to SARS‐CoV‐2 Variants in Australian Children,” Nature Communications 13, no. 1 (2022): 7185, 10.1038/s41467-022-34983-2. PMC9700848 36434068

[28] A. C. Dowell , T. Lancaster , R. Bruton , et al., “Immunological Imprinting of Humoral Immunity to SARS‐CoV‐2 in Children,” Nature Communications 14, no. 1 (2023): 3845, 10.1038/s41467-023-39575-2. PMC10310754 37386081

[29] V. Servellita , A. M. Syed , M. K. Morris , et al., “Neutralizing Immunity in Vaccine Breakthrough Infections From the SARS‐CoV‐2 Omicron and Delta Variants,” Cell 185, no. 9 (2022): 1539–1548.e5, 10.1016/j.cell.2022.03.019. 35429436 PMC8930394

[30] C. He , X. He , J. Yang , et al., “Spike Protein of SARS‐CoV‐2 Omicron (B.1.1.529) Variant Have a Reduced Ability to Induce the Immune Response,” Signal Transduction and Targeted Therapy 7, no. 1 (2022): 119, 10.1038/s41392-022-00980-6. 35397623 PMC8994023

[31] M. Baz , N. Deshpande , C. Mackenzie‐Kludas , F. Mordant , D. Anderson , and K. Subbarao , “SARS‐CoV‐2 Omicron BA.1 Challenge After Ancestral or Delta Infection in Mice,” Emerging Infectious Diseases 28, no. 11 (2022): 2352–2355, 10.3201/eid2811.220718. 36191630 PMC9622237

[32] L. Wang , W. Shi , M. G. Joyce , et al., “Evaluation of Candidate Vaccine Approaches for MERS‐CoV,” Nature Communications 6 (2015): 7712, 10.1038/ncomms8712. PMC4525294 26218507

[33] L. Du , C. Ma , and S. Jiang , “Antibodies Induced by Receptor‐Binding Domain in Spike Protein of SARS‐CoV Do Not Cross‐Neutralize the Novel Human Coronavirus hCoV‐EMC,” Journal of Infection 67, no. 4 (2013): 348–350, 10.1016/j.jinf.2013.05.002. 23685240 PMC7127489

[34] N. Wang , J. Shang , S. Jiang , and L. Du , “Subunit Vaccines Against Emerging Pathogenic Human Coronaviruses,” Frontiers in Microbiology 11 (2020): 298, 10.3389/fmicb.2020.00298. 32265848 PMC7105881

[35] S. Sun , J. He , L. Liu , et al., “Anti‐S2 Antibodies Responsible for the SARS‐CoV‐2 Infection‐Induced Serological Cross‐Reactivity Against MERS‐CoV and MERS‐Related Coronaviruses,” Frontiers in Immunology 16 (2025): 1541269, 10.3389/fimmu.2025.1541269. 40226608 PMC11985752

[36] J. Hicks , C. Klumpp‐Thomas , H. Kalish , et al., “Serologic Cross‐Reactivity of SARS‐CoV‐2 With Endemic and Seasonal Betacoronaviruses,” Journal of Clinical Immunology 41, no. 5 (2021): 906–913, 10.1007/s10875-021-00997-6. 33725211 PMC7962425

[37] C. L. Hsieh , A. P. Werner , S. R. Leist , et al., “Stabilized Coronavirus Spike Stem Elicits a Broadly Protective Antibody,” Cell Reports 37, no. 5 (2021): 109929, 10.1016/j.celrep.2021.109929. 34710354 PMC8519809

[38] S. H. Kim , Y. Kim , S. Jeon , et al., “Rise in Broadly Cross‐Reactive Adaptive Immunity Against Human β‐Coronaviruses in MERS‐Recovered Patients During the COVID‐19 Pandemic,” Science Advances 10, no. 9 (2024): eadk6425, 10.1126/sciadv.adk6425. 38416834 PMC10901372

[39] H. T. Zedan , M. K. Smatti , S. Thomas , et al., “Assessment of Broadly Reactive Responses in Patients With MERS‐CoV Infection and SARS‐CoV‐2 Vaccination,” JAMA Network Open 6, no. 6 (2023): e2319222, 10.1001/jamanetworkopen.2023.19222. 37389876 PMC10314312

[40] S. Lusvarghi , S. D. Pollett , S. N. Neerukonda , et al., “Oward SARS‐CoV‐2 Omicron Neutralization by Therapeutic Antibodies, Convalescent Sera, and Post‐mRNA Vaccine Booster,” bioRxiv 12, no. 22 (2021): 473880, 10.1101/2021.12.22.473880.

[41] M. Y. Khan , N. D. Aljehani , R. H. Abdulal , et al., “Intranasal CD40‐targeted Recombinant MERS‐CoV S1 Protein Is Superior to Intramuscular Immunization in Eliciting Systemic and Mucosal Immune Responses in Mice,” Vaccine 69 (2026): 127975, 10.1016/j.vaccine.2025.127975. 41232171

[42] Y. Sun , Q. Li , Y. Luo , et al., “Development of an RBD‐Fc Fusion Vaccine for COVID‐19,” Vaccine: X 16 (2024): 100444, 10.1016/j.jvacx.2024.100444. 38327768 PMC10847155

